# Application of nnU-Net for Automatic Segmentation of Lung Lesions on CT Images and Its Implication for Radiomic Models

**DOI:** 10.3390/jcm11247334

**Published:** 2022-12-09

**Authors:** Matteo Ferrante, Lisa Rinaldi, Francesca Botta, Xiaobin Hu, Andreas Dolp, Marta Minotti, Francesca De Piano, Gianluigi Funicelli, Stefania Volpe, Federica Bellerba, Paolo De Marco, Sara Raimondi, Stefania Rizzo, Kuangyu Shi, Marta Cremonesi, Barbara A. Jereczek-Fossa, Lorenzo Spaggiari, Filippo De Marinis, Roberto Orecchia, Daniela Origgi

**Affiliations:** 1 Medical Physics Unit, IEO European Institute of Oncology IRCCS, via Ripamonti 435, 20141 Milan, Italy; 2 Radiation Research Unit, IEO European Institute of Oncology IRCCS, via Ripamonti 435, 20141 Milan, Italy; 3 Department of Informatics, Technical University of Munich, Arcisstraße 21, 80333 Munich, Germany; 4 Division of Radiology, IEO, European Institute of Oncology IRCCS, via Ripamonti 435, 20141 Milan, Italy; 5 Division of Radiation Oncology, IEO European Institute of Oncology IRCCS, via Ripamonti 435, 20141 Milan, Italy; 6 Department of Oncology and Hemato-Oncology, University of Milan, via Festa del Perdono 7, 20122 Milan, Italy; 7 Department of Experimental Oncology, IEO European Institute of Oncology IRCCS, via Ripamonti 435, 20141 Milan, Italy; 8 Clinica di Radiologia EOC, Istituto Imaging della Svizzera Italiana (IIMSI), via Tesserete 46, 6900 Lugano, Switzerland; 9 Faculty of Biomedical Sciences, Università della Svizzera Italiana (USI), via G. Buffi 13, 6900 Lugano, Switzerland; 10 Chair for Computer-Aided Medical Procedures, Department of Informatics, Technical University of Munich, Arcisstraße 21, 80333 Munich, Germany; 11 Department of Nuclear Medicine, Bern University Hospital, University of Bern, Freiburgstrasse 18, 3010 Bern, Switzerland; 12 Division of Thoracic Surgery, IEO, European Institute of Oncology IRCCS, via Ripamonti 435, 20141 Milan, Italy; 13 Division of Thoracic Oncology, IEO, European Institute of Oncology IRCCS, via Ripamonti 435, 20141 Milan, Italy; 14 Scientific Direction, IEO, European Institute of Oncology IRCCS, via Ripamonti 435, 20141 Milan, Italy

**Keywords:** nnU-Net, NSCLC, automatic segmentation, radiomics, hand-crafted/deep features, predictive model

## Abstract

Radiomics investigates the predictive role of quantitative parameters calculated from radiological images. In oncology, tumour segmentation constitutes a crucial step of the radiomic workflow. Manual segmentation is time-consuming and prone to inter-observer variability. In this study, a state-of-the-art deep-learning network for automatic segmentation (nnU-Net) was applied to computed tomography images of lung tumour patients, and its impact on the performance of survival radiomic models was assessed. In total, 899 patients were included, from two proprietary and one public datasets. Different network architectures (2D, 3D) were trained and tested on different combinations of the datasets. Automatic segmentations were compared to reference manual segmentations performed by physicians using the DICE similarity coefficient. Subsequently, the accuracy of radiomic models for survival classification based on either manual or automatic segmentations were compared, considering both hand-crafted and deep-learning features. The best agreement between automatic and manual contours (DICE = 0.78 ± 0.12) was achieved averaging 2D and 3D predictions and applying customised post-processing. The accuracy of the survival classifier (ranging between 0.65 and 0.78) was not statistically different when using manual versus automatic contours, both with hand-crafted and deep features. These results support the promising role nnU-Net can play in automatic segmentation, accelerating the radiomic workflow without impairing the models’ accuracy. Further investigations on different clinical endpoints and populations are encouraged to confirm and generalise these findings.

## 1. Introduction

In Europe, lung cancer is the second most common malignancy in men and the third most common in women with higher incidence rates in developed over undeveloped countries. Non-small cell lung cancer (NSCLC) accounts for 80–90% of lung malignancies, and includes adenocarcinoma, squamous cell carcinoma and large cell carcinoma [1,2].

The usual diagnostic pathway for lung cancer is based on computed tomography (CT) scan of the head, thorax and superior abdomen (to include the adrenals), and PET-CT. The histological confirmation of the presence of malignancy is usually achieved by CT-guided biopsy, when the lesion is in the outer part of the lung, or by fine needle aspiration under endoscopic and ultrasound guidance, when the lesion is in the inner part of the lungs. The latter is also used in cases of positive lymph nodes at PET-CT, to confirm the presence of nodal involvement [3,4].

After diagnosis, the treatment of these malignancies can involve different clinical pathways.

Advanced methods can be applied to the radiological images, especially CT, to derive useful information for the physicians in order to improve diagnostic accuracy and choose the best treatment for each patient [5,6]. Among these radiomics, the calculation of a wide amount of quantitative parameters from medical images has shown promising results for the prediction of therapy outcomes, survival probabilities and other clinical endpoints, including tumour type, stage, mutation status and presence of metastasis [7,8,9,10,11,12]. In addition, artificial-intelligence (AI) -based methodologies have been increasingly used, either within the radiomic workflow or alone [13,14,15,16,17,18,19]. The final aim of these approaches is to mine high-level information from radiological images, which are not visible to the human eye but might be relevant for clinical purpose. Such methodologies are rapidly emerging as complementary to, and in some cases replacing, the previous approaches used for lung image analysis, including the use of semantic features [20] and of computer-aided diagnosis tools [21]. In particular, AI-based techniques appear promising for overcoming the main issues preventing the clinical implementation of the previous and current methodologies, mainly represented by lack of standardisation, generalisability [6,22] and the time resources that would be required for their use in the daily routine.

The current traditional radiomic approach is based on the calculation of descriptors, named radiomic features, from the numerical content of the medical images. These descriptors quantify different properties of the area of the image under investigation, among them shape, signal intensity and texture, and can be analysed for possible association with clinical endpoints using different methodologies, including statistical approaches or machine-learning AI techniques [23]. In the most advanced AI applications, such descriptors can be learnt by deep-learning algorithms rather than being calculated with hand-crafted tools [24,25,26,27].

One of the main bottlenecks of radiomic studies is the segmentation of the volume of interest (VOI), typically the lesion in oncological applications. This is a crucial step, since all subsequent operations of the radiomic workflow (feature extraction and model development) are concerned with the VOI only. Segmentation is most often performed manually by one or more expert physicians, and it is a very demanding task, especially for being extremely time-consuming. For this reason, semi-automatic techniques were introduced in the field of medical segmentation in the past decades [28,29,30]. In these approaches, even though most of the segmentation is performed by an algorithm, human intervention is still required to guide the procedure. As a result, in addition to needing time from a dedicated resource, the semi-automatic segmentation techniques might suffer from an important limitation of the fully manual contouring: the inter- and intra-reader variability [30,31,32,33,34]. AI can play a role in this context as well, with the introduction of fully automatic techniques [35,36]. Once conveniently trained and validated, such algorithms can drastically reduce the time spent by radiologists in monitoring lesion segmentation, and at the same time would allow the number—and therefore the reliability—of the datasets under investigation to be increased.

Automatic segmentation has in recent years also been investigated for the contouring of lung tumours. In particular, deep-learning algorithms were extensively adopted in the segmentation field [18,37,38,39] because of their flexibility and generalisability. Most studies focused on different variants of the basic architecture U-Net, a famous convolutional neural network (CNN) that down-samples and up-samples image features sharing information between the two processes at each level [35]. Among these, nnU-Net is currently considered the state-of-the-art framework; it includes ad-hoc automated pre-processing and outperforms most of the other variants in a wide variety of experiments [40]. In addition, nnU-Net is available online, offering the possibility to test it, train it on wider datasets and under different configurations, ultimately promoting generalisability.

The present study fits into this scenario and has two purposes.

The first aim was to train different configurations of nnU-Net on a wide, real-world dataset of CT images acquired from lung cancer patients at different stages in order to identify the configuration with the highest accuracy in comparison to the manual segmentation performed by physicians, taken as reference standard.

The second aim was to assess to what extent the use of automatic segmentation, as a replacement for manual contouring, affects the performance of a radiomic predictive model. To this purpose, radiomic models for the prediction of five-years survival were built on a population of early-stage NSCLC patients, comparing the performances obtained when extracting hand-crafted or deep radiomic features from manual versus automatic segmentation.

## 2. Materials and Methods

### 2.1. Patient Population and CT Acquisition

In this study, three different datasets of patients diagnosed with NSCLC were analysed, two retrospectively collected at the European Institute of Oncology (Milano, Italy)—dataset A and dataset B—and one publicly available, dataset C.

Patients included in datasets A and B were staged before pre-treatment by a contrast-enhanced CT scan and a PET-CT, according to the current guidelines. More specifically, dataset A included 270 patients staged up to pT3pN1M0, undergoing surgery soon after acquisition of a diagnostic contrast-enhanced CT at the European Institute of Oncology without pre-operative chemotherapy. The clinical characteristics of this population have been described elsewhere [10]. The information on the acquisition parameters of the CT image can be found in the Supplementary Materials—Table S2.

Dataset B included 217 patients extracted from a database of 261 patients affected by advanced NSCLC, undergoing chemotherapy after the acquisition of a diagnostic contrast-enhanced CT image. Clinical characteristics of this population are reported in Supplementary Materials—Table S1, while CT acquisition information is listed in Supplementary Materials—Table S2. Only patients undergoing CT imaging at the European Institute of Oncology were selected.

The Institutional Review Board of the European Institute of Oncology approved this retrospective study (UID-2078), waiving the need for informed consent.

Dataset C included 412 patients extracted from the *Lung1* public dataset [41] of the NSCLC-Radiomics collection on The Cancer Imaging Archive platform (TCIA) [42]. The full dataset is composed of 422 patients; of these, 10 were excluded due to metadata instability or corrupted labels (i.e., clashes between the images and segmentations in eight cases, respiratory motion artefacts in two cases). Clinical information can be retrieved from [43] and the TCIA website (https://wiki.cancerimagingarchive.net/display/Public/NSCLC-Radiomics, accessed on 1 July 2021).

### 2.2. Segmentation

#### 2.2.1. Manual Segmentation

One lesion per patient was segmented on the axial CT images by manually delineating the border of the lesion slice by slice.

Datasets A and B were contoured by three radiologists with different degrees of experience, after a common agreement on the procedure. The common criteria among radiologists included the adoption of both the lung and the mediastinal visualisation windows (width of 1500 HU and level of −600 HU and width of 350 HU and level of 40 HU, respectively) according to the lesion location and to the contrast with the surrounding tissues; moreover, vessels were excluded and opacity along the lesion edges was included. Segmentation was performed on the AWServer platform (v. 3.2 Ext. 2.0 tool, GE Healthcare, Chicago, IL, USA) and saved in RT structure format.

Original segmentations from dataset C segmentations were downloaded from the online archive and imported on 3D Slicer version 4.10.2 (NIH, Bethesda, MD, USA) [44]. Each segmentation was revised by a radiation oncologist, who edited the volume of interest as needed. Specifically, all nodal areas were excluded from the gross tumour volume to overcome possible inconsistencies between features extracted from the primary tumour and regional lymph nodes. Blood vessels were excluded, as well.

The final masks were then saved in nearly raw raster data (nrrd) format.

#### 2.2.2. Automatic Segmentation: Training and Testing

The nnU-Net, previously developed for automatic segmentation tasks in medical imaging [40], was adopted for the present study. Based on a U-Net architecture, it represents a state-of-the-art framework, improving on previous ones by introducing empirical rules for image pre-processing which allow for the achievement of high performance in the segmentation task. The net is trained by providing a set of images (training set) as input along with the corresponding manual segmentation performed by the physicians, considered as the reference standard to be learnt. During the training phase, the parameters of the network are learnt in order to produce automatic segmentations as similar as possible to the manual reference segmentation. Then, in inference, it is tested on previously unseen images (test set) for which the network provides the automatic segmentation as output without receiving the manual segmentation in input.

The model training and testing was repeated in twelve different configurations to investigate the variation of the model performance with different source data and network architecture. The configurations were chosen by using different combinations of the three datasets (A, B and C) as training or test sets, and varying the spatial resolution of the images and of the manual segmentation masks. The split between training and test sets was performed randomly, without overlaps between them to avoid overfitting. This procedure aimed to increase the intrinsic variability of the tested datasets, and to identify the model yielding the best performance in terms of computing power, training timing and segmentation performance. Images and masks were used either at full resolution (512 × 512) or reduced to half spacing (256 × 256) using the SimpleITK Python library for voxel resampling [45]. Resampling was used to assess whether the computation time and memory footprint could be lightened without losing performances. As soon as more computational power became available during the study, we opted for an approach without a-priori resampling, referring for this step to the specific architecture chosen (fullres or lowres) which internally already performs these operations.

In Table 1, all the configurations investigated in this study are listed along with the description of the training modality (described in detail in the following), the initial image resolution, and the number of cases used for training and testing.

Regarding training modality, at least two different models (architectures) were trained for each configuration, and the performance of the ensemble model was evaluated. The ensemble configuration combines the outputs of multiple architectures by averaging, for each voxel, the probabilities of belonging to the lesion instead of the background predicted by each architecture. Except for configuration #3, an ensemble (2D, 3D fullres) approach was used, which combines a 2D and a full resolution (fullres) 3D U-Net architecture. In the case of 2D architecture, the training is performed by taking each slice separately as input and using a 2D convolutional kernel. In the 3D architecture, the CT images are analysed considering also the adjacent slices with 3D kernels in order to catch the volumetric (inter-slices) information and thus provide the learning process with the maximum context. In configuration #3, on the other hand, a cascade approach was applied. The cascade architecture consists first of all of a 3D U-Net, trained using down-sampled inputs; the outputs of the segmentation are then up-resampled to the original size and used as additional inputs for another 3D U-Net, which is trained at full resolution. This approach was tested in one configuration only, due to the intensive requirements in terms of computational resources.

In all cases, the network was trained using the nnU-Net “all” flag, except in the case of the “cascade” model. For the latter, the training was repeated five times by operating a five-fold cross validation. This made it possible to test numerous configurations in acceptable times by measuring the variations of the performance.

More information about the nnU-Net parameters and available architecture modalities can be found in the Supplementary Materials (Additional information on the segmentation network (nnU-Net)).

A customised post-processing algorithm—different from the one proposed in the original nnU-Net paper—was developed for the present study and applied to the output of the network. In its original version, the nnU-Net package applies a post-processing, returning only the largest connected component with a non-maxima suppression algorithm. We calculated, instead, the connected components in three dimensions using the Python connected components 3D library (https://pypi.org/project/connected-components-3d/#description, accessed on 7 September 2021) to individuate and separate all the segmented regions, in this way taking into account cases in which the network identifies multiple lesions or false positives. In particular, we separated each connected volume estimated as a lesion and computed its volume. Estimates of the ensemble and 3D approaches were proposed with decreasing confidence in order of volume size, so that it was possible to choose the most suitable segmentation, even when multiple lesions were identified, and discard false positives.

**Figure 1:**
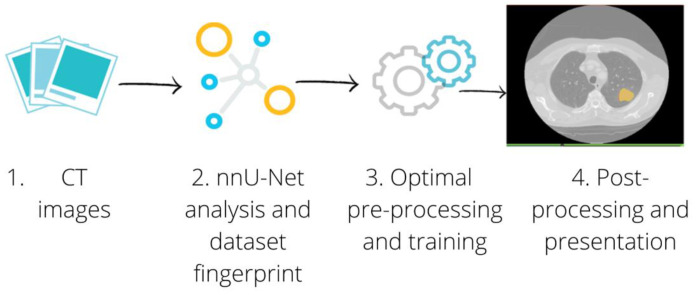
Segmentation Pipeline. The CT images of patients with a lung lesion were collected, along with the manual contours used as the ground truth (**1**). For each configuration we separated the cases into training and test set. The training set was passed through the nnU-Net framework (**2**), which automatically adapted the pre-processing to perform the optimal training (**3**). We finally applied the post-processing to the best performing model to present each connected component provided by the network separately (**4**).

A pipeline of the segmentation procedure is shown in Figure 1.

The segmentation network was trained for a minimum of 400 epochs, defined as a 250 batch size as in the original nnU-Net paper on a server with a Nvidia RTX 2080 Ti GPU Card with 11 GB of dedicated RAM memory. Each training modality required between 10 and 20 h to be completed.

#### 2.2.3. Automatic Segmentation Performance

The performance of the automatic segmentation pipeline on each testing dataset was assessed by computing the DICE similarity coefficient, a measure of the degree of volume overlapping between the manual (ground truth) and the automatic segmentation produced by the network [46]. It ranges between 0, when the two segmentation masks do not share common voxels, and 1, when they are perfectly overlapped.

In addition, the percentage of lesions correctly identified by the network was assessed by calculating the number of lesions with a DICE greater than 0 out of the total number of cases in each test set. Similarly, the percentage of lesions with good and excellent correspondence with the ground truth was assessed by calculating the number of lesions with a DICE greater than 0.5, and greater than 0.8 from the total cases in each test set (the values being arbitrarily chosen with reference to the mean DICE value obtained in the different configurations).

### 2.3. Survival Prediction

Radiomic models for classifying patients according to survival were performed on dataset A, extracting radiomic features from either manual or automatic segmentation, with hand-crafted and deep-learning methodologies, as detailed in the next paragraphs. To this purpose, the automatic contours generated by training the nnU-Net on datasets B and C and by testing on dataset A were adopted (configuration #12 in Table 1). The reason for choosing this configuration was related to the availability of survival follow-up data for patients in dataset A.

Two additional steps were included in the segmentation pipeline: first, among all the identified connected components, the one with the highest DICE, meaning the highest overlap with the ground truth, was selected. Then, all the cases for which the DICE coefficient was lower than 0.3 were excluded from the analysis [47]. These two steps, applied taking advantage of the availability—in this development phase—of the ground truth manual segmentation for all patients, were intended to simulate the role of the physician in a real clinical application of the automatic algorithm, where manual segmentation is not available. In this latter case, indeed, the physician is expected to revise the segmentations proposed by the automatic algorithm, rejecting the cases for which the segmented object is not the lesion (step 1) and the cases for which the lesion is correctly identified by the automatic algorithm, but contoured in a bad way (the concept of bad contour being translated, in terms of DICE score, as: DICE score < 0.3, step 2 [47]). For this reason, hereafter we will refer to this refinement operation as a “radiologist simulation” procedure.

#### 2.3.1. Hand-Crafted Radiomic Features Extraction

Hand-crafted features were extracted from both the manual and the automatic contours using the open-source tool Pyradiomics (v. 2.2.0) [48]. The features were extracted from the “original” images, meaning that no filter was applied to the CT image before extraction. Moreover, we analysed only features extracted in 2D from each axial slice and averaged among all the slices of the mask, as recommended when the voxels are not isotropic (https://arxiv.org/pdf/1612.07003.pdf).

As part of the pre-processing techniques, pixel size in the axial plane was resampled (“sitkBSpline” interpolation, the default in Pyradiomics), and the grey-level intensities were discretised to a fixed bin width of 25 HU (Hounsfield Units).

The categories of features analysed in this study were: shape, first order, grey level co-occurrence matrix (glcm), grey level run length matrix (glrlm), grey level size zone matrix (glszm), neighbouring grey tone difference matrix (ngtdm) and grey level dependence matrix (gldm). A total of 165 “original” features were available within Pyradiomics. Among these, 12 features were discarded, either because they were intrinsically dependent on the number of voxels inside the VOI or because they actually coincided with other features for the chosen configuration/parameters. More details on the reasons for exclusion, and the full list of the 153 included features can be found in the Supplementary Materials in [49].

For each feature, the interclass correlation coefficient (ICC) [50] was calculated to compare the values extracted from manual versus automatic contours. The ICC ranges between 0 and 1, indicating, respectively, no or perfect agreement between measurements. The *irr* package (v. 0.84.1) in R was used to calculate the ICC. The analysis was also stratified for different DICE coefficient intervals: [0.30, 0.50), [0.50, 0.70), [0.70, 0.80), [0.80, 0.90) and [0.90, 1.00], in order to investigate the dependence of features agreement in relation to the contours’ agreement.

#### 2.3.2. Deep-Learning Feature Extraction

A pipeline involving a neural network was developed to select and extract features autonomously in order to compare or integrate the traditional hand-crafted radiomic approach with a deep one.

Due to the limited size of dataset A, a transfer-learning approach was adopted, relying on models pre-trained for more general purposes.

More precisely, a pre-processing pipeline was developed using the Medical Open Network for AI library (MONAI) [51]. First, the image was resampled at a fixed size of 1 × 1 mm in the axial plane and 1.5 mm in the perpendicular plane (spline interpolation), and then it was cut to a fixed size of 224 × 224 × 152, centred at the centre of the segmented volume, in order to be compatible with the chosen network architecture. Subsequently, the ACSConv library [52], based on PyTorch [53,54], was used to transform a pre-trained 2D model into a 3D model. Basically, the 2D convolutional layers were replaced with their three-dimensional versions by processing the 2D weights to derive the corresponding 3D version, resulting in a three-dimensional pre-trained model, particularly suitable in medical images for being focused on the processing of axial, coronal and sagittal views.

The ResNet152 model [55] was finally applied to extract 2048 deep features from the latest AdaptiveAvgPool3d layer of the PyTorch implementation.

#### 2.3.3. Survival Model Implementation

Survival characteristics of the dataset A population, updated in October 2021, were collected. In particular, for each patient, the survival expressed in the months after the date of the CT examination was recorded. Based on this information, patients were dichotomised according to 5-year survival [56], and the possibility for predicting the survival class (below/above 5 years) based on radiomic features was investigated using machine-learning (ML) techniques. The radiomic features used as input variables were either the hand-crafted features, deep features, or hybrid features (the latter being a combination of hand-crafted and deep features obtained by concatenating these two groups of features), extracted from either manual or automatic contours. This analysis made it possible to investigate to what extent the use of manual or automatic segmentation can affect the performance of this kind of radiomic-based dichotomous survival model.

A preliminary analysis was performed in order to compare different ML algorithms, and different combinations of hyperparameters for each algorithm, with the purpose of identifying the algorithm and hyperparameters with the best model performance (cross-validation). This preliminary phase involved only features extracted from manual segmentation, since it represents the gold standard segmentation in this study; it considered hand-crafted, deep and hybrid features separately. The investigated algorithms were: Random Forest (RF), Support Vector Machine (SVM) and Multilayer Perceptron models (MLP); all of them incorporate a feature selection step to identify the features mostly associated with the outcome. For each model, 50 hyperparameter combinations were considered. To this purpose, the Weight & Biases (W&B) [57] Python library was used.

Subsequently, once the best ML model and the best combination of hyperparameters were identified according to an accuracy metric, the selected ML model was trained for each of the six cases under analysis: using hand-crafted features, deep features or hybrid features, extracted from either manual or automatic contours, as the input.

Finally, a ten-fold cross validation was performed on the entire dataset in order to obtain a statistic measure of the accuracy. The accuracy results of the ten-fold cross validation for the six cases were compared using a t-test to check if the distributions were statistically different.

In this way, the impact on model performance of using manual versus automatic segmentation and hand-crafted radiomic features versus deep features versus hybrid features was assessed.

**Figure 2:**
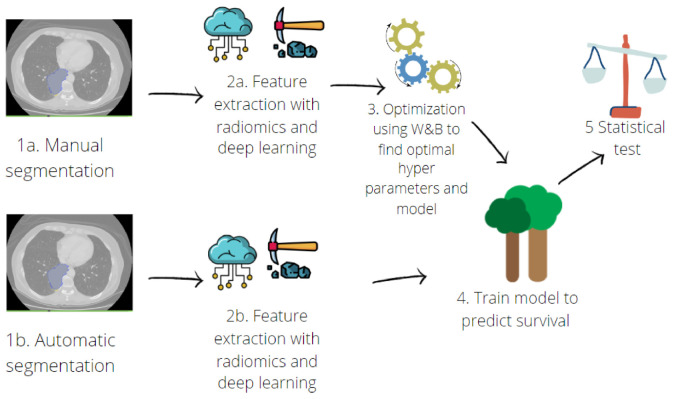
Pipeline for predictive model implementation. Hand-crafted and deep features were extracted (**2a**) from manual contours (**1a**). Then, an optimisation step was performed (**3**) with a random search across the hyperparameters of three different machine learning algorithms: Random Forest, Support Vector Machine and Multilayer Perceptron. Once the best model was found, deep-learning and radiomic features were also extracted from contours obtained with automatic segmentation (**1b**,**2b**). The predictive model was trained (**4**) on features extracted from both manual and automatic segmentation, and a ten-fold cross validation was performed. Finally, performances were compared using a *t*-test (**5**).

The pipeline for the survival model implementation is schematised in Figure 2.

The entire code, including the segmentation tool, was developed with Python v 3.8 using popular deep-learning and machine learning libraries, including PyTorch [53,54], MONAI [51] and Scikit-Learn [58].

## 3. Results

### 3.1. Manual and Automatic Segmentation

According to the manual segmentation, mean lesion volume was 38.5 cm^3^ (range 0.2–511.9 cm^3^) for dataset A, 57.2 cm^3^ (range 0.1–708.3 cm^3^) for dataset B, and 68.2 cm^3^ (range 0.5–648.9 cm^3^) for dataset C.

The performance of the automatic segmentation pipeline without applying any post-processing is shown in Table 2 for each of the configurations investigated, listed in Table 1. The mean DICE coefficient (± standard deviation) and the percentage of lesions correctly identified using the automatic tool (DICE > 0) are reported, along with the percentage of lesions with a good (>0.50) and excellent (>0.80) DICE value. The mean DICE value is calculated considering only the outputs of the network with a DICE > 0. The network correctly identified (DICE > 0) from 82% up to 94% of lesions, depending on the configuration, with an average DICE among all configurations equal to 0.70 (min = 0.65, max = 0.77). For the majority of lesions, the automatic contours achieved a DICE value larger than 0.50 in all configurations (range: 68–82%); a DICE over 0.80 was obtained for less than 50% of the lesions in almost all configurations.

The inclusion of dataset C in the pipeline reduced the segmentation performance in terms of percentage of correctly identified lesions. However, the performance in terms of average DICE was similar to the configurations without dataset C. On the other hand, adding a completely independent cohort—as dataset C—made it possible to increase the intrinsic variability of the training set, and thus the generalisability of the network.

**Figure 3:**
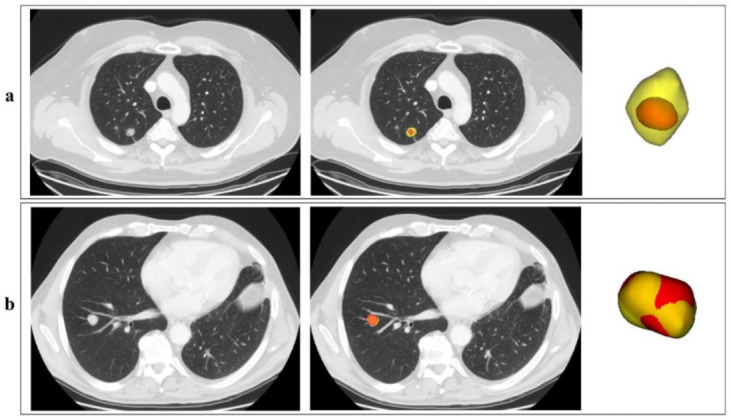
Examples of the automatic segmentation outputs. In the picture, two outputs of the segmentation network, corresponding to two different patients (**a**,**b**), are reported in red, superimposed onto the manual segmentation in yellow. For each patient, one axial CT slice including the lesion is reported, without (**left**) and with (**right**) indication of the lesion contours; the 3D visual representation of the contours is also reported. Although the two lesions are quite similar in shape and volume, the lesion for patient in Figure 3a is characterised by a low-density edge which is not captured by the automatic algorithm.

Two examples of the output of the segmentation pipeline are reported in Figure 3, with automatic segmentation displayed in red, superimposed onto the manual segmentation (ground truth) displayed in yellow. Figure 3a shows a case with a low DICE (0.43), meaning a not-optimal overlap between the automatic segmentation and the manual one. Figure 3b is instead an example of an excellent DICE (0.88).

**Figure 4:**
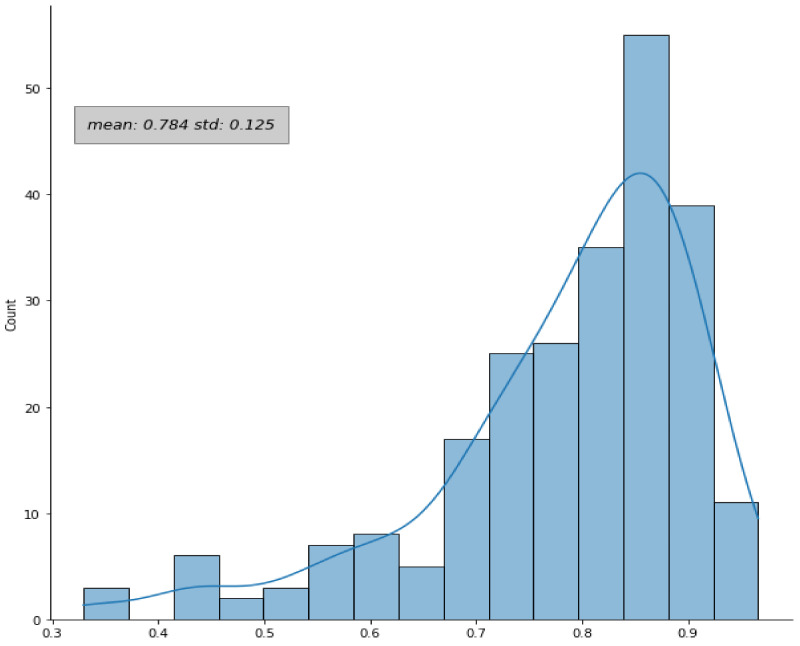
Distribution of the DICE coefficient. The DICE coefficients were evaluated for the patients in dataset A by comparing the automatic and the manual contours in configuration #12. The plot refers to the results after the application of the post-processing and the “radiologist simulation” steps.

In the case of configuration #12 (training on datasets B and C, testing on dataset A, used for the subsequent survival analysis), when the post-processing and the “radiologist simulation” procedure were added, an improvement of the DICE coefficient was observed: 90% (242/270) of the lesions had a DICE coefficient greater than 0.3, with a mean DICE equal to 0.78 ± 0.12 (compared to 0.72 ± 0.29 without any post-processing). The distribution of the DICE coefficient over the dataset A in this configuration is shown in Figure 4.

To further check the segmentation performance, the volume of the predicted segmentation (V_automatic_) was compared to the ground truth (V_manual_) by computing the ratio V_automatic_/V_manual_; a mean value of 1.04 was obtained, indicating that on average the predictions were just 4% apart in terms of volume, with respect to the manual reference.

When comparing the radiomic features extracted from manual versus automatic contours, we observed that 11% (17/153) and 22% (34/153) of the hand-crafted features had an excellent (ICC ≥ 0.90) and a good (0.75 ≤ ICC < 0.90) agreement between manual and automatic contours. The majority of the features (47%), instead, had a moderate agreement (0.50 ≤ ICC < 0.75), and the remaining 19% (29/153) had an ICC value lower than 0.50 (poor agreement).

When analysing the agreement separately in each DICE coefficient range ([0.30, 0.50), [0.50, 0.70), [0.70, 0.80), [0.80, 0.90), [0.90, 1.00]), an improvement of the overall ICC was observed for the group of patients with higher DICE coefficient values. In general, for each category of features analysed, a trend was observed with the ICC increasing as a function of the DICE coefficient. A graphical overview of this behaviour is reported in the Supplementary Materials—Figure S1. Nonetheless, some features might exhibit poor or good agreement irrespective of the DICE value.

### 3.2. Survival Model

As previously reported, when the post-processing and the “radiologist simulation” procedure were applied to configuration #12, 242 patients from dataset A had a valid segmentation; they were all included in the survival prediction analysis. A total of 132 patients had a survival longer than 5 years, and the remaining 110 fell in the class with survival below 5 years.

Using the W&B library to keep track of the dependence of the hyperparameters on the three different ML models tested, the best performances were achieved with RF. During the optimisation phase, the *n_estimates* (number of trees in the RF model) and a low value of *ccp_alpha* (regularisation parameter for the cost-complexity pruning algorithm) were positively correlated with accuracy in the training set. As a result, a RF classifier with 1000 trees and a ccp_alpha of 0.01 were selected for the implementation of the classification model, which was trained six times in the six different cases according to the type of features (handcrafted/deep/hybrid) and to the type of contour used for feature calculation.

In Table 3, the accuracy of the model achieved using hand-crafted features, deep features, and a combination of them (hybrid) is reported, separately for features calculated on manual or automatic contours.

**Figure 5:**
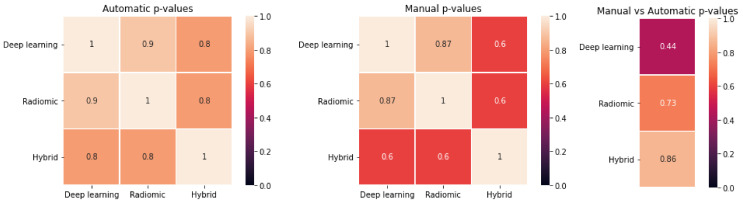
*T*-test results of the comparison between the different approaches. (**Left**): *p*-values for deep-learning versus hand-crafted versus hybrid features for automatic segmentation. (**Centre**): *p*-values for deep-learning versus hand-crafted versus hybrid features for manual segmentation. (**Right**): *p*-values for manual versus automatic in case of deep-learning, hand-crafted and hybrid features.

The *p*-values of the t-test comparing the accuracies obtained from ten-fold cross validation on the entire dataset are reported in Figure 5. No statistical difference was observed (all *p* > 0.05) between the model accuracy obtained with hand-crafted, deep or hybrid features, for both manual and automatic contours, nor when comparing manual versus automatic contours for a given group of features (hand-crafted, deep or hybrid).

## 4. Discussion

In recent years, many studies and methodologies have emerged with the aim of extracting quantitative information from medical images, potentially relevant to predict clinical outcomes. In the near future, some of these might prove their usefulness in simplifying and guiding choices towards personalised treatments. However, to be efficiently integrated into the clinical practice, besides being robust and generalisable they need to be easily implementable on a routine basis.

Segmentation is in most cases an unavoidable and crucial step of these procedures. Automatic algorithms capable of producing reliable segmentations in a short time could drastically reduce the time required for clinicians for this type of activity, allowing access to much larger segmented datasets during the model development phase. In this regard, it might not be crucial to train automatic algorithms segmenting the region of interest with equal accuracy as the manual gold standard. Indeed, the identification of algorithms segmenting the region of interest with enough accuracy to produce comparable predictive models as those obtained with manual segmentation might equally serve the purpose.

In this study, a pipeline based on a state-of-the-art framework, nnU-Net, was developed for automatic segmentation of NSCLC tumours using CT images, testing different network architectures on different dataset configurations. The ability to perform a pre-processing aimed at and suited to the characteristics of a CT thoracic image dataset was exploited, and a post-processing algorithm was optimised for lung lesion segmentation. The automatic contours were compared with manual ones, drawn by experienced physicians. A survival predictive model was then built, based on radiomic features calculated from either manual or automatic segmentations, and the model performances were compared. In addition, we investigated the difference in model performance for the clinical outcome under investigation, when using hand-crafted radiomic features, deep-learning features, or a hybrid approach.

Concerning the automatic segmentation procedure, good results were obtained both for the volume of overlap between the ground truth and the output of the network (evaluated with the DICE coefficient) and for the number of correctly detected lesions. The average DICE among all the dataset configurations was equal to 0.70 and the percentage of correctly identified lesions was higher than 82% in all the investigated cases (Table 2).

These results are largely in agreement with others reported in the literature. Gan et al. [59] created a combination of a 3D and 2D network to segment lung lesions using a total of 260 patients from a private dataset, achieving a mean DICE coefficient of 0.72 ± 0.10. Yang et al. [52] developed an ACS (axial–coronal–sagittal) network and tested it on the LIDC–IDRI dataset of lung nodules [60]. The main idea behind this architecture was to use 2D kernels on the three views separately and then combine them to obtain a 3D output. The best DICE was obtained on the ACS pretrained model architecture, and it was equal to 0.76, outperforming both the 3D (best DICE equal to 0.75) and 2D (DICE equal to 0.69) pretrained networks. Better results were obtained by Haarburger et al. [47]. In this study, the authors applied a probabilistic segmentation algorithm based on a 2D U-Net, named PHiSeg network [61], to three datasets of different tumours (lung, kidney and liver lesions) and obtained a DICE metric of 0.85 (IQR between 0.77 and 0.89) for the LIDC–IDRI lung dataset.

As previously anticipated, a perfect agreement between different segmentations (DICE equal to 1) might not be fundamental for the purpose of predictive model creation. An automatic segmentation not entirely matching the manual gold standard contour but leading to predictive models with comparable accuracy to those built on manual contours reaches the scope of overcoming the manual contouring limitations, without impairing the final goal for the clinics. We tested this for a specific clinical endpoint, namely the classification of patients according to survival.

To this purpose, one of the configurations analysed in the segmentation pipeline was selected, the one using datasets B and C for training and dataset A for testing.

As a first step, in this specific configuration, the customised post-processing was applied to the masks obtained from the segmentation network, improving the DICE coefficient in the subset of selected lesions from 0.72 ± 0.29 to 0.78 ± 0.12.

The predictive models obtained using hand-crafted, deep or hybrid features calculated from manual or automatic segmentations had comparable accuracy (Table 3). Although the models built on automatic contours provided slightly higher accuracy than those obtained on manual contours (0.78 versus 0.65–0.73), and deep features appeared to perform worse than hand-crafted features in the manual setting (0.65 versus 0.73), a non-significant difference was found (*t*-test *p*-values > 0.05) between the performances obtained during a ten-fold cross validation with the two types of segmentation and the three categories of extracted features (Figure 5).

In the case of hand-crafted features, the comparable models’ accuracy was achieved although the differences between the features extracted from manual versus automatic contours were in some cases not negligible, especially for contours exhibiting low DICE coefficient values (Figure S1 in Supplementary Materials).

This was a relevant result proving that a perfect agreement between manual and automatic contours, and the corresponding radiomic features, might not be necessary for the purpose of radiomic-based predictive model creation. This might be related to the fact that while the disagreement between manual and automatic contours occurs mostly on the lesion edge, the features relevant for the clinical outcome of interest might be those capturing the properties of the internal voxels, affected to a lower extent by the outer voxels. Nonetheless, it should be noted that, despite the features’ agreement generally increasing when the contours’ agreement does, some exceptions might occur, with some features exhibiting good or bad agreement irrespective of the DICE value. In this regard, it might be interesting to repeat the comparison between the performances of manual versus automatic predictive models separating the patients according to the value of DICE coefficient. However, it must be pointed out that this result was obtained for the specific clinical endpoint considered in this study (dichotomous classification of survival) and it was tested on a single, multicentre sample. Generalisability of this finding to other clinical outcomes (e.g., mutational status), or other populations, will be properly investigated in dedicated datasets; the same populations will be used as additional, independent test sets in order to further assess the segmentation performance. In this study, multiple physicians were involved in the contouring of the lung lesions for the three datasets used in the segmentation pipeline. Even if common criteria were established among them, some variability may have been introduced, reducing the segmentation performance of the network. On the other hand, the presence of multiple operators performing reference, manual segmentation in the training set could also represent an advantage for algorithm generalisability. Further studies will be mandatory for the evaluation of the inter-reader variability in manual segmentation, and its impact on the training of automatic tools. Among other limitations, a relatively small number of images was used for the development of the survival model. Larger datasets might allow us to obtain superior performance. However, in this study we did not focus on the validation of a survival model. Rather, our primary goal was the comparison of the model performance in different scenarios (segmentation modality and/or feature type) when the same group of patients was involved.

## 5. Conclusions

An automatic tool for lung tumour segmentation in CT images was adopted based on the nnU-Net framework and properly adapted with customised post-processing. After testing different network architectures on multiple datasets, the best model achieved an average DICE coefficient of 0.78 ± 0.12 after the application of ad hoc post-processing techniques, correctly finding and segmenting 90% of the tested lesions.

The radiomic features extracted from thus-obtained automatic contours resulted in survival predictive models having comparable accuracy to the ones obtained extracting features from the reference manual contours (accuracy statistically not distinguishable). In addition, hand-crafted and deep radiomic features provided comparable results in terms of predictive accuracy with both segmentation modalities.

These findings support the idea that segmentation tools based on deep learning can be effectively included in the image analysis workflow, dramatically reducing the physician’s workload without impairing the accuracy in comparison to the use of manual segmentation. If confirmed on other populations and for other clinical endpoints, this could simplify access to large datasets and accelerate the identification of reliable tools and their translation to the clinical practice.

## Figures and Tables

**Table 1:** Investigated combinations of the three datasets for the training and testing of nnU-Net.

	Configuration ^		Training Modality	Initial Image Resolution	# pts Training	# pts Testing
#	Training Dataset	Testing Dataset				
1	A	A *	ensemble(2D, 3D fullres)	256 × 256	220	50
2	A + B	A + B *	ensemble(2D, 3D fullres)	256 × 256	296	128
3	A + B	A + B *	ensemble(cascade, 3D fullres)	512 × 512	340	147
4	C	C *	ensemble(2D, 3D fullres)	512 × 512	328	84
5	C	A	ensemble(2D, 3D fullres)	512 × 512	328	79
6	C	B	ensemble(2D, 3D fullres)	512 × 512	328	66
7	C	A + B	ensemble(2D, 3D fullres)	512 × 512	328	147
8	A + B + C	A	ensemble(2D, 3D fullres)	512 × 512	668	80
9	A + B + C	B	ensemble(2D, 3D fullres)	512 × 512	668	67
10	A + B + C	C	ensemble(2D, 3D fullres)	512 × 512	668	84
11	A + B + C	A + B + C *	ensemble(2D, 3D fullres)	512 × 512	668	231
12	B + C	A	ensemble(2D, 3D fullres)	512 × 512	629	270

^ The two “configuration” columns, one for the training and the other for the test sets, report the name of the dataset/datasets from which the images were extracted. * For this configuration, the training and test sets were created starting from the same dataset/datasets, but the patients used for the two sets were not the same, meaning that there was no overlap between the two groups. # stands for “number of”.

**Table 2:** Results of segmentation, before post-processing and including only the outputs with a DICE > 0. DICE is intended as mean ± standard deviation. The last three columns correspond to the number of lesions with a DICE > 0, DICE > 0.5 and DICE > 0.80, respectively, across the total number of test cases.

	Configuration ^		DICE	% correctly Identified (DICE > 0) Lesions	% Lesions with DICE > 0.50	% Lesions with DICE > 0.80
#	Training Dataset	Testing Dataset				
1	A	A *	0.65 ± 0.29	94%	74%	38%
2	A + B	A + B *	0.74 ± 0.28	93%	82%	51%
3	A + B	A + B *	0.66 ± 0.32	93%	71%	41%
4	C	C *	0.68 ± 0.33	86%	71%	32%
5	C	A	0.69 ± 0.33	83%	69%	35%
6	C	B	0.71 ± 0.32	85%	73%	40%
7	C	A + B	0.70 ± 0.33	84%	71%	37%
8	A + B + C	A	0.71 ± 0.32	88%	70%	48%
9	A + B + C	B	0.77 ± 0.31	87%	79%	48%
10	A + B + C	C	0.67 ± 0.32	83%	68%	31%
11	A + B + C	A + B + C *	0.71 ± 0.32	86%	72%	42%
12	B + C	A	0.72 ± 0.29	91%	78%	45%

^ The two “configuration” columns, one for the training and the other for the test sets, report the name of the dataset/datasets from which the images were extracted. * For this configuration, the training and test sets were created starting from the same dataset/datasets, but the patients used for the two sets were not the same, meaning that there was no overlap between the two groups.

**Table 3:** Accuracy results of the survival prediction using the RF model, comparing manual and automatic contours. The model performances are given for the three types of features extracted: hand-crafted features (extracted using conventional radiomics), deep-learning features (extracted using a deep-learning algorithm), and hybrid features (obtained by concatenating the first two types).

	Manual	Automatic
Hand-crafted features	0.73	0.78
Deep features	0.65	0.78
Hybrid features	0.70	0.78

## Data Availability

The radiomic data obtained for this study are available upon reasonable request to the corresponding author.

## Supplementary Materials

The following supporting information can be downloaded at: https://www.mdpi.com/article/10.3390/jcm11247334/s1, Table S1: Clinical characteristics collected for the 261 patients from which we extracted dataset B; Table S2 Acquisition information of the CT images of datasets A and B; Table S3 Parameters (fixed and rule-based parameters) used during the training of the nnU-Net network; Figure S1 Boxplot showing the ICC value according to the DICE range.

## Abbreviations

CT = computed tomography; NSCLC = Non-small Cell Lung Cancer; PET-CT = positron emission tomography—computed tomography; VOI = volume of interest; HU = Hounsfield unit; ML = machine learning; RF = Random Forest; SVM = Support Vector Machine; MLP = Multilayer Perceptron models; Std = standard deviation.
