# Supplementary material for: Application of nnU-Net for Automatic Segmentation of Lung Lesions on CT Images and Its Implication for Radiomic Models
Source: J Clin Med. 2022 Dec 9;11(24):7334. doi: 10.3390/jcm11247334 (PMC9784875; doi:10.3390/jcm11247334)
Supplement: Supplementary file 1 [file jcm-11-07334-s001.zip › jcm-2007426-supplementary.pdf]

Supplementary materials

MATERIALS AND METHODS

1. Additional information on the datasets

Clinical characteristics

Table S1 lists the clinical information for the entire population from which we extracted patients of dataset B.

Clinical parameters		Dataset
Number of patients		261
Age at CT (years) *		65.9 ±10.4
Nodule volume (cm3) *		57.23 ± 97.5
Gender	Female	97/261 (37%)
	Male	164/261 (63%)
Lobe	LID	41/257 (16%)
	LIS	96/257 (12%)
	LMD	8/257 (3%)
	LSD	87/257 (34%)
	LDD	80/257 (31%)
	MIXED	10/257 (4%)
Stage at diagnosis	Not IV	103/261 (39%)

IV	159/261 (61%)

Table S1 Clinical characteristics collected for the 261 patients from which we extracted datasets B.

*Mean value ± standard deviation

Acquisition characteristics

For Dataset A and Dataset B patients, CT images were acquired on one of the scanners available at our Institute (Lightspeed Ultra, Lightspeed 16, Optima 660 and Discovery 750 HD, all from GE Healthcare, Milwaukee, Wisconsin, USA) adopting the diagnostic thoracic protocol utilised in our Institute. The protocol included one series, selected for the present study, acquired during the portal phase after the injection of iodinated contrast medium. Contrast medium was one among the following: Visipaque® 320, GE Healthcare; Ultravist® 370, Bayer; Iomeron 350, Bracco Imaging; Xenetix 350, Guerbet. The injected amount depended on contrast medium type, concentration, and patient weight.

The images were acquired in helical mode, with 2.5mm slice thickness and slice spacing. The CT image were reconstructed with standard convolutional kernel and two different algorithms, filtered backprojection (FBP) and iterative reconstruction (Adaptive Statistical Iterative Reconstruction, ASIR). The acquisition and reconstruction parameters which might affect image texture and hence the value of radiomic features for datasets A and B are included in **Table S2**.

Table S2 Acquisition information of the CT images of the dataset A and B.

Acquisition parameters		Dataset A	Dataset B
CT scanner	LightSpeed Ultra	52 (19%)	-
	LightSpeed 16	135 (50%)	-
	Optima CT660	69 (26%)	117 (54%)
	Discovery CT750 HD	14 (5%)	100 (46%)
Voltage peak	100	-	29 (13%)
	120	270 (100%)	176 (81%)
	140	-	12 (6%)
Exposure (mAs)		16 ± 10 (14)	9 ± 4 (7)
Pixel size (mm)		0.75 ± 0.07 (0.73)	0.78 ± 0.07 (0.77)
Slice thickness (mm)		2.5	2.5
Contrast medium	Iomeron	201 (74%)	7 (3%)
	Xenetix	8 (3%)	-
	Visipaque	28 (10%)	37 (17%)
	Ultravist	29 (11%)	166 (77%)
	n.k.	4 (2%)	7 (3%)
Reconstruction algorithm	FBP	187 (69%)	-
	IR	83 (31%)	217 (100%)

IR = iterative reconstruction; FBP = filtered backprojection; n.k. = not know

2. Additional information on the segmentation network (nnU-Net)

The network adopted for this study was the nnU-Net, a self-configuring pipeline developed by Isensee et al. [40], which is able to train the model without the need of complex manual intervention, such as hyperparameter optimisation or data preparation. Some information about the network characteristics is reported below.

During the training of the nnU-Net three classes of parameters are adjusted.
The first group determines the learning strategy (architecture, initial learning rate and its scheduling, loss function and metrics to be used, number of epochs and batch size), and the parameters' value is fixed regardless of the type of dataset.
The second group of parameters is set automatically based on the dataset characteristics using a set of empirical rules, derived from the literature and from experience in segmentation. They include intensity normalization, resampling strategy of the image and of the mask, network topology and use or not of instance normalization.
Finally, the third set of parameters is determined empirically on the dataset and regulates the choice of post-processing and model selection. For example, a cross-validation procedure can be applied in order to select the best model or a combination of models among those trained, using the DICE to evaluate the performance of each configuration. The type of models which can be trained are: 2D axial model, 3D full resolution, 3D low resolution, cascade. The **2D Axial** model is based on 2D U-Net architecture, trained considering each axial slice separately. The **3D full resolution** (fullres) model is a 3D U-Net based on 3D convolutions and trained using CT images at full resolutions, in order to provide maximum context for segmentations. Despite the advantage of high resolution and maximum available information, 3D fullres approach has a huge memory footprint and requires high computation capacity. For this reason, it is also possible to train a **3D low resolution** model, where images are downsampled to half or one quarter resolution. When large GPU memory is available the combination in **cascade** of the two models, low-resolution followed by high-resolution, usually outperforms the single application of each one. In this case, first a low-resolution model is trained, which outputs a low-resolution segmentation mask. Then, the full resolution CT and the low-resolution mask are used for the training of a second model which can take advantage of the

low-resolution mask provided by the first model which just need to be up-sampled and redefined using finer details, elaborated from full resolution images. As often happens in machine learning problems, averaging outputs of different models usually increases the metrics and produce better – and more reliable – results. Therefore, the **ensemble** approach can be adopted by averaging the response of any combination of two models.

Furthermore, it is possible to refine the result of the segmentation, via connected component selection. The main idea of this post-processing procedure consists in the exclusion of all the smaller components and the comparison of the results (with and without the application of the connected component selection) via cross-validation, using the DICE for the evaluation of the performance.

Details about the settings used for the purpose of this study are reported in **Table S3** for the first and second group of parameters. The third group of parameters (empirical parameters) was not used in the present study since ad-hoc post-processing procedure was developed to keep more control on the results.

Table S3 Parameters (fixed and rule-based parameters) used during the training of the nnU-Net network

**Fixed parameters:** set of parameters that are fixed by the framework for all experiments	Learning rate	Poly learning rate schedule (initial, 0.01)
	Loss function	Dice and cross-entropy
	Architecture template	Encoder–decoder with skip-connection ('U-Net-like') and instance normalization, leaky ReLU
	Optimizer	SGD with Nesterov momentum (μ = 0.99)
	Data augmentation	Rotations, scaling, Gaussian noise, Gaussian blur, brightness, contrast, simulation of low resolution, gamma correction and mirroring

**Rule-based parameters:** set of parameters for which the actual values are determined by the following rules and the values of the specific dataset	Intensity normalization Modality	global dataset percentile clipping & z score with global foreground mean and standard deviation. The images are clipped to 0.5 and 99.5 percentiles and then normalized using the z_score.
	Image resampling strategy	in-plane with third-order spline, out of-plane with nearest neighbour
	Image target spacing	lowest resolution axis tenth percentile, other axes median

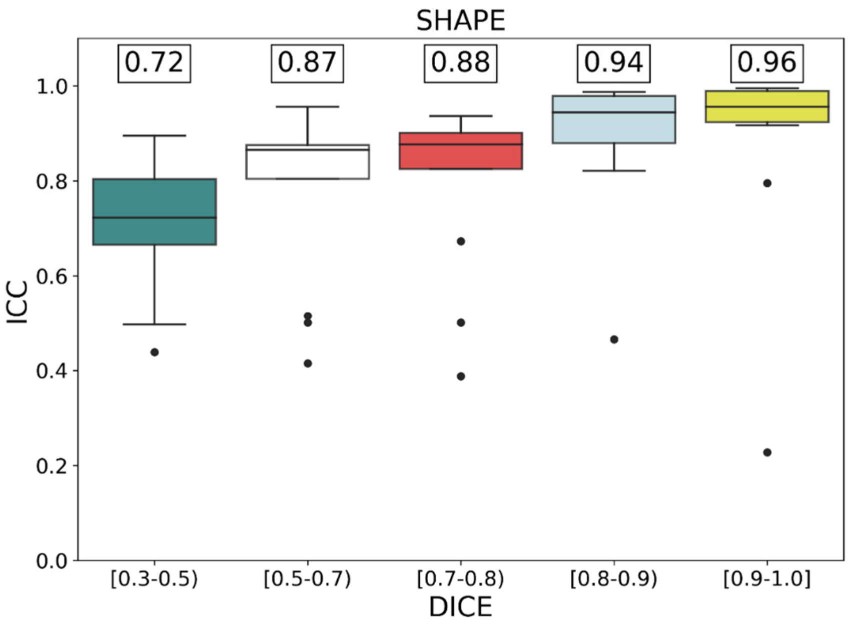

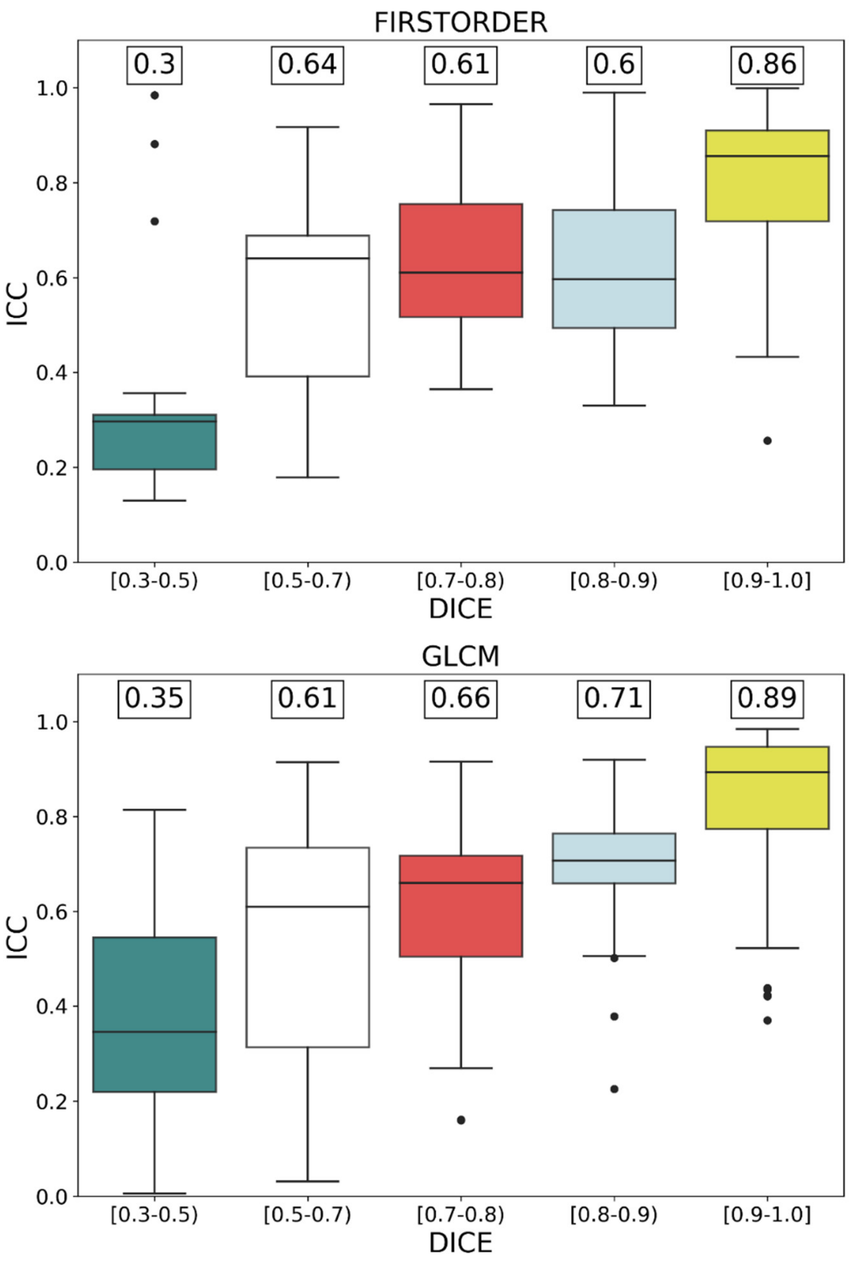
FIRSTORDER
0.3
0.64
0.61
0.6
0.86
ICC
DICE
[0.3-0.5)
[0.5-0.7)
[0.7-0.8)
[0.8-0.9)
[0.9-1.0]
GLCM
0.35
0.61
0.66
0.71
0.89
ICC
DICE
[0.3-0.5)
[0.5-0.7)
[0.7-0.8)
[0.8-0.9)
[0.9-1.0]

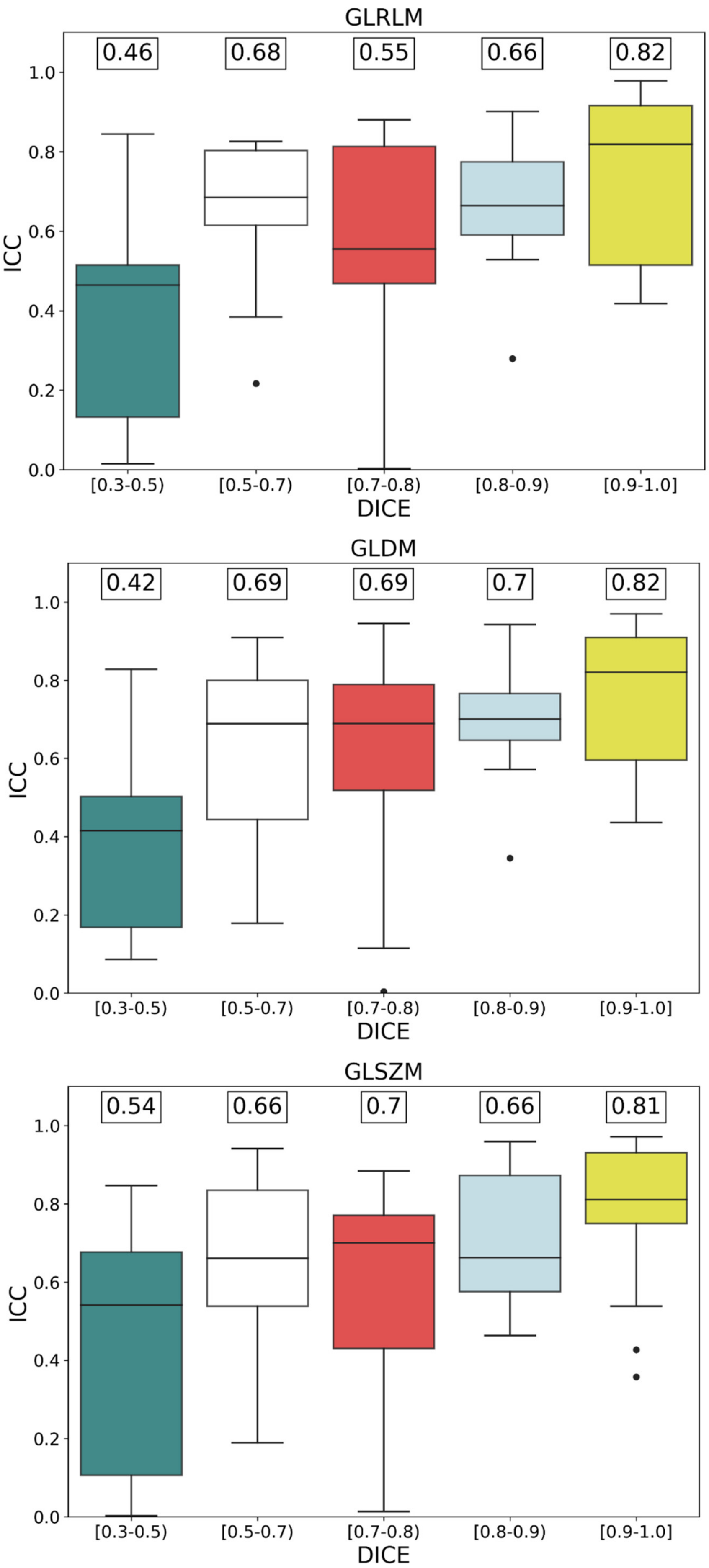
GLRLM
0.46
0.68
0.55
0.66
0.82
ICC
1.0
0.8
0.6
0.4
0.2
0.0
[0.3-0.5)
[0.5-0.7)
[0.7-0.8)
[0.8-0.9)
[0.9-1.0]
DICE
GLDM
0.42
0.69
0.69
0.7
0.82
ICC
1.0
0.8
0.6
0.4
0.2
0.0
[0.3-0.5)
[0.5-0.7)
[0.7-0.8)
[0.8-0.9)
[0.9-1.0]
DICE
GLSZM
0.54
0.66
0.7
0.66
0.81
ICC
1.0
0.8
0.6
0.4
0.2
0.0
[0.3-0.5)
[0.5-0.7)
[0.7-0.8)
[0.8-0.9)
[0.9-1.0]
DICE

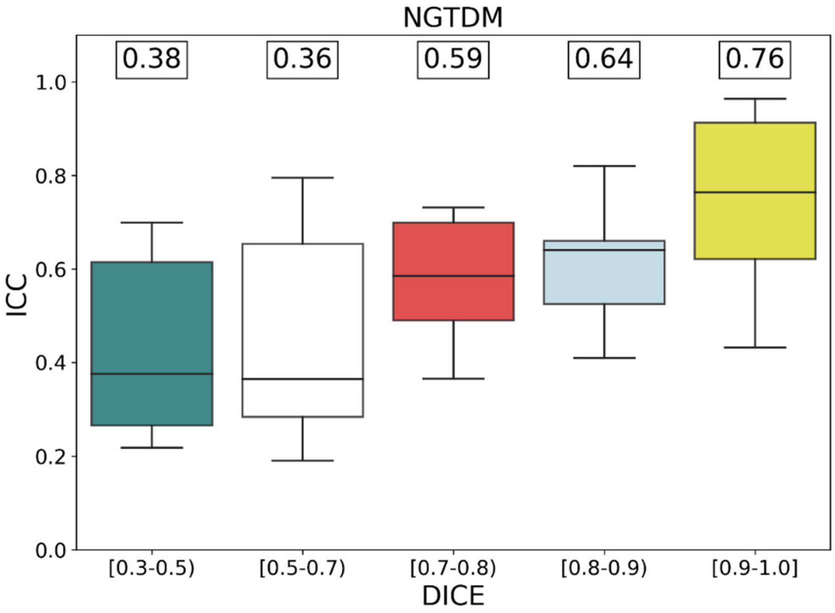

Figure S1 Boxplot showing the ICC value according to the DICE range. Each plot corresponds to a different category of hand-crafted radiomic features (shape, first order, glcm, glrlm, gldm, glszm, ngtdm). The median value of the ICC for each DICE range is reported above each box.
